# Associations of novel serum lipid index with epithelial ovarian cancer chemoresistance and prognosis

**DOI:** 10.3389/fonc.2023.1052760

**Published:** 2023-02-13

**Authors:** Yuan Li, Chunliang Shang, Huamao Liang, Kun Zhang, Yu Wu, Hongyan Guo

**Affiliations:** Department of Obstetrics and Gynecology, Peking University Third Hospital, Beijing, China

**Keywords:** serum lipids, epithelial ovarian cancer, chemoresistance, prognosis, novel serum lipid index

## Abstract

**Purpose:**

To evaluate the relationship between novel serum lipid index and chemoresistance as well as prognosis of epithelial ovarian cancer (EOC).

**Patients and methods:**

Patients’ serum lipid profiles of 249 cases diagnosed with epithelial ovarian cancer, including total cholesterol (TC), high-density lipoprotein cholesterol (HDL-C), low-density lipoprotein cholesterol (LDL-C) as well as their ratios, the novel indicators HDL-C/TC and HDL-C/LDL-C, and clinicopathologic characteristics were retrospectively collected and calculated from January 2016 to January 2020 and correlation between serum lipid index and clinicopathological features such as chemoresistance as well as prognosis were evaluated.

**Results:**

249 patients pathologically diagnosed EOC who underwent cytoreductive surgery were included in our cohort. The mean age of these patients was 55.20 ± 11.07 years. Binary logistic regression analyses indicated Federation International of Gynecology and Obstetrics (FIGO(stage and HDL-C/TC ratio had significant association with chemoresistance. Univariate analyses demonstrated pathological type, chemoresistance, FIGO stage, neoadjuvant chemotherapy, maintenance treatment, HDL-C/LDL-C ratio, HDL-C/TC ratio were related to Progression-Free Survival (PFS) and Overall Survival (OS) (P<0. 05). Particularly, multivariate analyses indicated that HDL-C/LDL-C ratio was independent protective factors for both PFS and OS.

**Conclusion:**

The complex serum lipid index HDL-C/TC ratio has a significant correlation with chemoresistance. HDL-C/LDL-C ratio is closely related to the clinicopathological characteristics and prognosis of patients with EOC and is an independent protective factor indicating better outcome.

## Introduction

Ovarian cancer (OC) is the most lethal malignant tumor of female reproductive system. The standard treatment for ovarian cancer is cytoreductive surgery combined with platinum-based chemotherapy and maintenance therapy based on tumor heterogeneity (1, 2). Lack of sensitive and effective screening methods, as well as atypical clinical symptoms, leads to initial diagnosis of advanced stage in more than 70% of patients. Although the initial response rate is 70–80% including 40 to 50% complete response, majority of patients relapse within 2 years with subsequent resistance to chemotherapy, leading to a 5-year survival rate of ovarian cancer patients only 45% with a gradually shortened interval between recurrences (3).

Multidisciplinary management of ovarian cancer patients is key to improve outcome. The introduction of imaging biomarkers, such as those obtained with FDG PET/CT, could help improve patient outcome, by precisely staging and restaging, as well as providing a prognostic value (4). More serological markers have also become a hotspot in predicting tumor prognosis.

Disturbance of lipid metabolism in cancer cells can lead to structural changes of tumor cell membrane, turbulence of intracellular energy metabolism, dysregulation of cell signal transduction and gene expression (5). As the source and transforming component of lipid metabolism in tumor microenvironment, serum lipids play an important role in tumorigenesis. A greater synthesis or absorption of lipids has been found in a variety of cancers as the raw materials contributing to the growth of cancer cells and the formation of tumors. Reprogramming of lipid metabolism can also affect the development of tumor cells by changing the fluidity of cell membranes and weakening immune function (6). It is reported that serum lipid levels are closely related to the occurrence and development of malignant tumors such as breast cancer (7), gastric cancer (8) and lung cancer (6). Mesenchyme-derived adipocytes in the tumor microenvironment can promote tumorigenesis of prostate cancer by releasing fatty acids and related factors (9). Adipose stem cells derived from the human omentum can promote proliferation, migration, chemoresistance of ovarian cancer cells and radiation resistance in nude mice model of ovarian cancer (10). There is also a correlation between lipid metabolism in tumor microenvironment and the efficacy of targeted drugs. For example, adipose abundance in breast cancer tumors was significantly correlated with resistance to trastuzumab (11).

The main components of serum lipids are total cholesterol (TC), high density lipoprotein cholesterol (HDL-C) and low-density lipoprotein cholesterol (LDL-C). TC is an essential lipid to maintain cell homeostasis and an important part of cell membrane, which is rich in lipid raft and plays a key role in intracellular signal transduction (12). HDL-C maintains normal cellular cholesterol homeostasis by removing excess cholesterol from cells and transporting it to the liver. When the level of HDL-C is low, the accumulated cholesterol is utilized by cancer cells for membrane formation, thereby promoting the development of cancer. On the contrary, LDL-C is a significant carrier of cholesterol from the liver to tissues throughout the body, providing energy and materials for tumor cell proliferation and development (12). It has been reported in literatures, TC、TG、HDL-C and LDL-C are independent factors for survival prediction of various tumors such as gastric cancer (13) and breast cancer (14).

Novel complex indicators of lipid metabolism, such as TG/HDL-C ratio, have been proved superior to that of single TG or HDL-C level in predicting survival of patients with triple negative breast cancer (15) and gastric cancer (7). These results suggest that complex novel indicators might be better predictive index for survival prediction. Correlation between complex lipid indicators and prognosis in patients of ovarian cancer has not been clearly reported. Therefore, this study aimed to explore the correlation between serum lipid levels and chemoresistance as well as prognosis in patients of ovarian cancer.

## Material and methods

### Patients

**Figure 1:**
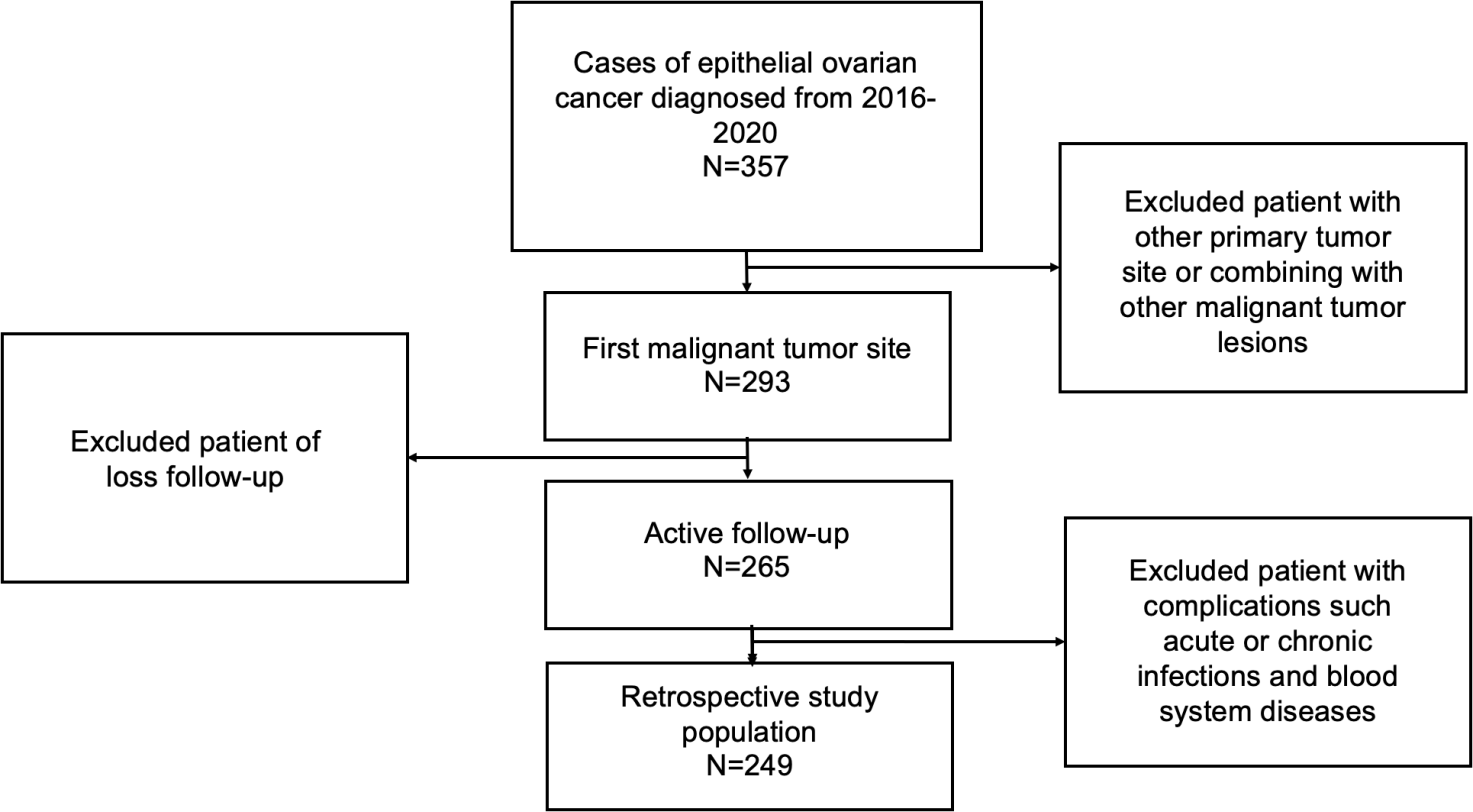
Workflow for selection procedure of epithelial ovarian cancer patients.

249 patients pathologically diagnosed EOC who underwent cytoreductive surgery at the Department of Obstetrics and Gynecology, Beijing University Third Hospital between January 2016 to January 2020 were retrospectively reviewed in our cohort. Patients were included if they: ① pathologically confirmed to be EOC; ② ovary is the primary lesion of tumor; ③ received cytoreductive surgery combined with regular platinum-based chemotherapy in Peking University Third Hospital; ④ had complete preoperative peripheral blood data and complete follow-up; ⑤ There were no acute or chronic infections and blood system diseases, and not complicated with other tumors (**Figure 1** shows the workflow for selection procedure of epithelial ovarian cancer patients). This study was ratified by the Ethics Committee of Peking University Third Hospital. Written informed consent was obtained from all the patients in this study.

### Follow-up

Patients’ age, Federation of International of Gynecologists and Obstetricians (FIGO) stage, pathological type, tumor grade, therapeutic regimen and complications were collected from clinical attendance records. Follow-up began on the day of surgery and was conducted through direct telecommunications and outpatient visits until October 2021. The median follow-up time was 35 months. Follow-up of patients included blood tests, urine tests, computed tomography, and physical examination. Overall survival (OS) is measured from the date of surgery to the date of death from any cause, or the date of last follow-up visit. Progression-free survival (PFS) is identified from the date of surgery to the date of recurrence which was confirmed by imaging evidence or serum CA125 elevation. Platinum-sensitive is defined when patients have a clear response to the initial platinum-based treatment and achieve clinical remission, and progress or recurrence occurs more than 6 months (including 6 months) after the withdrawal of the previous platinum-containing chemotherapy. Platinum-resistant is defined when patients respond to initial chemotherapy but progresses or relapses occurs within 6 months of completion of chemotherapy.

### Statistical analysis

Differences of clinicopathological parameters and relative prognostic parameters between groups were evaluated by independent-samples Student’s t-test or Fisher’s exact test according to the distribution of data, and the normal distribution continuous variables were divided into groups by the median. Chi square test and binary logistic regression analyses were used to analyze the relationship between serum lipid indicators and chemoresistance as well as other clinicopathological characteristics of patients. The survival curves were calculated using the Kaplan–Meier method. Survival analyses were conducted by Cox proportional hazards model. All reported p-values were two-sided. P<0.05 was considered significant, and 95% CIs were calculated. All analyses were performed using the SPSS Statistics version 26.0 (IBM Corporation, Armonk, NY, USA).

## Results

### Baseline clinicopathological characteristics

The mean age of these patients was 55.20 ± 11.07 years. There were 161 patients with FIGO stage III to IV, accounting for 64.66%of the total number. 222 (89.16%) patients were diagnosed of high-grade EOC. Serous carcinoma was found in 191 cases (76.71%), and other pathological types of epithelial ovarian cancer together were 58 cases (23.29%). There were 56 patients (22.49%) who received neoadjuvant chemotherapy. 57 patients (22.89%) received maintenance therapy after surgery. Among 249 patients included in this study, a total of 70 patients (28.11%) had the clinical characteristic of chemoresistance after chemotherapy. Recurrence occurred in 120 patients (48.20%) and 55patients (22.09%) died during the following.

### Grouping serum lipid single and complex indicators by median

We divided normal distribution continuous variables TC, HDL-C, LDL-C, HDL-C/LDL-C, HDL-C/TC into low- or high-level groups by median. With TC’s median of 4.65mmol/L as the boundary, 126 patients were in the low TC group and 123 patients in high TC group. Grouped by HDL-C ‘s median of 1.10mmol/L, there were 127 patients in the low HDL-C group and 122 patients in the high HDL-C group. Divided by the LDL-C’s median of 2.90mmol/L, there were 126 patients in the low LDL-C group and 123 patients in the high LDL-C group. The median of normal distribution continuous variable HDL-C/LDL-C was 0.39, 125 patients were in the low HDL-C/LDL-C group and 124 patients in the high HDL-C/LDL-C group. There were 124 patients in the low HDL-C/TC group and 125 patients in the high HDL-C/TC group with the HDL-C/TC’s median of 0.24 as the boundary.

### Correlation between clinical features and serum blood lipid levels

**Table 1** shows the correlation between clinical features and serum blood lipid levels. Patients of higher TC groups tend to be suffered from serous ovarian cancer. Lower HDL-C/LDL-C groups were associated with advanced FIGO stage and having complication of diabetes mellitus.

**Table 1:** Correction between clinicopathological features and different lipid level groups of 249 EOC patients.

	High TC group (*n*=123)	Low TC group (*n* = 146)	*χ* ^2^	*P*	High HDL-C group (*n=*122)	Low HDL-C group (*n* = 127)	*χ* ^2^	*P*	High LDL-C group (n=123	Low LDL-C group (n = 126)	*χ* ^2^	P	High HDL-C/LDL-C group (*n* = 124)	Low HDL-C/LDL-C group (*n* = 125)	*χ* ^2^	*P*	High HDL-C/TC group (n = 125)	Low HDL-C/TC group (n = 124)	*χ* ^2^	*P*
Age			0.202	0.65			0.646	0.09			0.894	0.66			0.906	0.34			2.507	0.11
Pathological type																				
serous	87	104	4.857	0.03	88	103	0.603	0.09	91	100	0.315	0.74	96	95	0.07	0.79	97	94	0.122	0.74
non-serous	36	22			34	24			32	26			28	30			28	30		
FIGO stage																				
I- II	50	38	2.998	0.08	55	33	0.002	0.43	45	43	0.898	0.69	53	35	5.92	0.02	52	36	4.303	0.04
III- IV	73	88			67	94			78	83			71	90			73	88		
Tumor grade																				
high	113	109	1.851	0.17	109	113	0.926	1.04	112	110	0.341	0.06	108	114	1.084	0.3	109	113	0.994	0.32
medium/low	10	17			13	14			11	16			16	11			16	11		
chemoresistance																				
sensitive	88	91	0.014	0.91	90	89	1.201	0.52	87	92	0.893	0.69	93	86	1.184	0.28	95	84	2.101	0.15
resistance	35	35			32	38			36	34			31	39			30	40		
Diabetes mellitus																				
Yes	22	23	0.006	0.94	108	96	0.008	0.4	22	23	0.975	0.94	16	29	4.457	0.04	15	30	0.012	0.43
No	101	103			14	31			101	103			108	96			110	94		
Hypertension																				
Yes	40	53	2.422	0.12	40	53	0.681	0.15	40	53	0.664	0.06	42	51	1.277	0.26	42	51	0.219	0.72
No	83	73			82	74			83	73			82	74			83	73		

**Figure 2:**
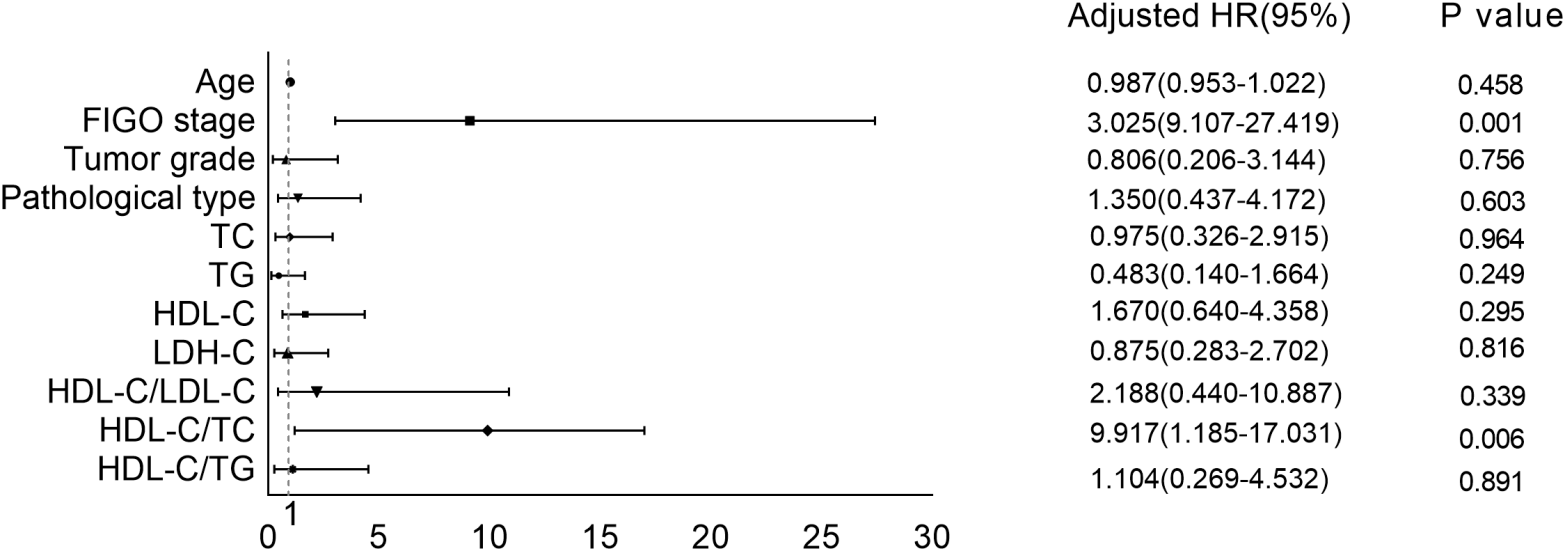
Correction between serum lipid levels as well as other clinical features and chemoresistance.

**Figure 2** shows binary logistic regression analyses indicating FIGO stage and HDL-C/TC ratio had significant correction with chemoresistance. Advanced FIGO stage and lower HDL-C/TC ratio mean a tendency to chemoresistance.

### Kaplan–Meier survival curves of patients with ovarian cancer stratified by HDL-C/LDL-C and HDL-C/TC ratios

**Figure 3:**
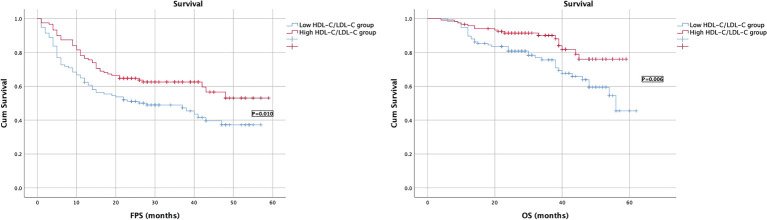
Survival curves of PFS and OS in EOC patients in low/high HDL-C/LDL-C groups.

**Figure 4:**
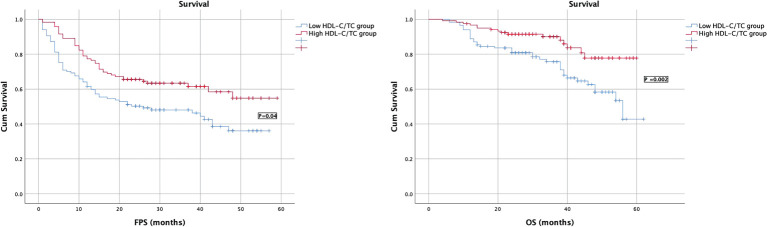
Survival curves of PFS and OS in EOC patients in low/high HDL-C/TC groups.

Kaplan–Meier survival analysis showed that patients of higher HDL-C/LDL-C and HDL-C/TC group had longer PFS and OS than those of lower HDL-C/LDL-C group, and the difference was statistically significant (P ≤ 0. 05), as shown in **Figures 3**, **4**.

### Univariate and multivariate analysis of FPS and OS

The survival analysis of PFS showed that the patient’s age, FIGO stage, pathological type, chemoresistance, primary treatment, maintenance therapy, HDL-C/LDL-C ratio, HDL-C/TC ratio, and whether having complication of diabetes mellitus were related to PFS (P<0. 05). Cox multivariate analysis showed that chemoresistance, FIGO stage, maintenance therapy, HDL-C/LDL-C ratio, and having complication of diabetes mellitus were independent predictive factors for PFS (P<0. 05), as shown in **Tables 2**, **3**.

**Table 2:** Univariate survival analysis of FPS in 249 patients with ovarian cancer.

Characteristics	Univariate analysis		
	P-value	HR	95%CI
**Age**	0.03	1.02	1.052-1.037
**Pathological type**	0.01	2.45	1.462-4.105
**FIGO stage**	0.01	3.63	2.285-5.764
**Tumor grade**	0.22	1.50	0.784-2.862
**chemoresistance**	0.01	0.09	0.058-0.131
**Primary treatment**	0.01	2.18	1.483-3.212
**maintenance therapy**	0.01	2.82	1.923-4.139
**TC**	0.53	1.06	0.886-1.265
**TG**	0.15	1.19	0.942-1.502
**HDL-C**	0.08	0.59	0.330-1.066
**LDL-C**	0.34	1.11	0.900-1.361
**HDL-C/LDL-C**	0.01	0.55	1.375-5.172
**HDL-C/TC**	0.01	1.66	0.369-0.807
**Diabetes mellitus**	0.04	1.56	1.019-2.380
**Hypertension**	0.33	0.83	0.570-1.206
**CA125**	0.38	1.00	1.000-1.000

**Table 3:** Multivariate survival analysis of FPS in 249 patients with ovarian cancer.

Characteristics	Multivariate analysis		
	P-value	HR	95%CI
**Age**	0.22	1.10	0.993-1.032
**Pathological type**	0.34	0.76	0.423-0.711
**FIGO stage**	0.01	0.42	0.243-0.711
**chemoresistance**	0.01	12.18	7.799-19.026
**Primary treatment**	0.82	0.95	0.621-1.455
**maintenance therapy**	0.02	0.62	0.414-0.935
**HDL-C/LDL-C**	0.01	5.21	1.661-16.33
**HDL-C/TC**	0.06	0.00	0.001-1.231
**Diabetes mellitus**	0.01	0.57	0.371-0.889

The survival analysis of OS showed that FIGO stage, pathological type, chemoresistance, primary treatment, maintenance therapy, HDL-C/LDL-C ratio, HDL-C/TC ratio, and whether having complication of diabetes mellitus were related to OS (P<0. 05). Cox multivariate analysis showed that chemoresistance, FIGO stage, maintenance therapy, and HDL-C/LDL-C ratio were independent predictive factors for PFS (P<0. 05), as shown in **Tables 4**, **5**.

**Table 4:** Univariate survival analysis of OS in 249 patients with ovarian cancer.

Characteristics	Univariate analysis		
	P-value	HR	95%CI
**Age**	0.09	1.02	0.996-1.048
**Pathological type**	0.01	0.33	0.143-0.781
**FIGO stage**	0.01	0.08	0.024-0.245
**Tumor grade**	0.46	0.71	0.283-1.784
**Chemoresistance**	0.01	9.61	4.401-17.101
**Primary treatment**	0.01	0.38	0.221-0.662
**Maintenance therapy**	0.01	0.48	0.273-0.851
**TC**	0.53	1.09	0.837-1.413
**TG**	0.32	1.19	0.845-1.666
**HDL-C**	0.07	0.44	0.181-1.055
**LDL-C**	0.13	1.27	0.936-1.718
**HDL-C/LDL-C**	0.01	0.55	1.375-5.172
**HDL-C/TC**	0.01	1.66	0.369-0.807
**Diabetes mellitus**	0.01	0.54	0.351-0.824
**Hypertension**	0.47	0.87	0.598-1.267
**CA125**	0.26	0.71	1.135-2.432

**Table 5:** Multivariate survival analysis of OS in 249 patients with ovarian cancer.

Characteristics	Multivariate analysis		
	P-value	HR	95%CI
**Pathological type**	0.71	0.33	0.143-0.781
**FIGO stage**	0.00	0.08	0.024-0.245
**chemoresistance**	0.00	9.61	4.401-17.101
**Primary treatment**	0.41	0.38	0.221-0.662
**maintenance therapy**	0.67	0.48	0.273-0.851
**HDL-C/LDL-C**	0.01	0.55	1.375-5.172
**HDL-C/TC**	0.97	1.66	0.369-0.807
**Diabetes mellitus**	0.10	0.69	0.447-1.076

## Discussion

In recent years, the relevance of lipid metabolism to the tumor microenvironment and cancer development has become a hot topic of research. To meet the demands of rapid growth, tumor cells try their best to obtain nutrients from outside, one of the main sources of energy is lipids in tumor microenvironment. At the same time, lipids are the main raw material of biofilm, which is important for the stability of cancer cell membrane and the reproduction and metastasis of cancers (16). Serum lipids are the main source of lipids in cancer microenvironment and are therefore closely related to the development of cancer (17). Studies have shown that in some solid tumors such as gastric cancer (18), lung cancer (19) and colon cancer (20), there are correlations between patients’ serum lipid levels and their clinical characteristics and prognosis. In the study of gynecological malignancies and lipid metabolism, researchers have focused on endometrial cancer (21, 22), which has a strong correlation between lipid metabolism and hormones. There has been no in-depth study of association between serum lipids and prognosis of ovarian cancer. Moreover, most of the studies on serum lipids and cancer just focus on single lipid index such as high/low density lipoproteins cholesterol and have not considered the combined effect of these indicators. It is therefore necessary to further explore the association between dyslipidemia and the clinical characteristics of ovarian cancer.

The main indicators of lipid metabolism in peripheral blood are total cholesterol, high-density lipoprotein cholesterol and low-density lipoprotein cholesterol. Cholesterol is an important component of the cell membrane and is essential for maintaining lipid raft stability which promotes cell proliferation (23). In the literature, patients of prostate cancer and cervical cancer with elevated total serum cholesterol levels have a poor prognosis (24, 25). Meanwhile, patients of non-small cell lung cancer, gastric cancer, and primary liver cancer with lower total serum cholesterol levels suggest a poor prognosis (11, 26, 27). A study of 229 patients with ovarian cancer and 233 patients with benign ovarian tumors showed a trend of lower total cholesterol levels in the ovarian cancer group compared to the benign ovarian tumor group, the hypothesis was that the rapid growth of cancer cells requires the involvement of large amounts of total cholesterol, which in turn promotes total cholesterol depletion (14, 28). However, no studies have reported a correlation between serum total cholesterol levels and the prognosis of ovarian cancer patients. In our research, we found that serum cholesterol levels correlated with the type of pathology of the patients, with serous type of ovarian cancer having higher cholesterol levels than other pathological types. High-density lipoprotein cholesterol is known as a vascular scavenger, which reduces cholesterol levels in cancer cells, peripheral tissues and tumor lessens biofilm raw materials by transporting cholesterol from surrounding tissues and converting it into bile acids for excretion from the intestine, which may antagonize the function of intracellular cholesterol in cancer cells to some extent. It has been reported that elevated high density lipoprotein level in peripheral blood is an independent prognostic factor for patients with gastric liver cancers. Zhang D. found that lower levels of HDL were an independent risk factor for the development of ovarian cancer (29). In contrast, LDL is primarily responsible for the distribution of cholesterol between extrahepatic tissues and cells, and elevated serum LDL level can lead to increased levels of cholesterol stored in cancer cells, thereby promoting cell proliferation. Andrew J. retrospectively analyzed serum lipid levels and disease-specific survival in 132 patients with epithelial ovarian cancer and concluded that serum LDL levels were negatively associated with patient disease-specific survival (30). Our study analyzed the relationship between serum HDL, LDL as well as cholesterol levels and the clinical characteristics and prognosis of patients with ovarian cancer and failed to obtain a statistical correlation.

Previous literatures as well as the results of our study reveal that there are few studies focusing on the prognostic evaluation of serum lipid levels in ovarian cancer and some conclusions are controversial. Therefore, we firstly introduced the previously reported high-density lipoprotein cholesterol/total cholesterol (HDL-C/TC) and high-density lipoprotein cholesterol/low-density lipoprotein cholesterol (HDL-C/LDL-C) as two composite lipid markers to better investigate association between serum lipid levels and clinical characteristics and prognosis of ovarian cancer. Previous studies have shown that in gastric cancer, HDL-C/TC ratio has better prognostic evaluation than a single HDL-C level (6). A similar study has demonstrated that TG/HDL was positively correlated with clinical features such as FIGO stage and pathological type of endometrial cancer (15). Our results show that HDL-C/LDL-C and HDL - C/TC ratios are negatively associated with patients’ FIGO stage. One of the mechanisms may be that a higher HDL-C/TC ratio increases the amount of cholesterol brought into the blood vessels by HDL thus reducing the amount of free cholesterol in cancer cells and weakening the ability of cancer cells to proliferate and metastasize. Using multiple regression analysis, we concluded that the HDL-C/TC ratio was negatively associated with chemoresistance in patients. It has been documented that cholesterol levels are elevated in the tumor microenvironment of ovarian cancer patients which can upregulate the expression of the drug efflux pump proteins ABCG2 and MDR1, as well as cholesterol receptor LXRα/β to reduce the sensitivity of ovarian cancer cells to chemotherapeutic agents (31).

In terms of prognosis, univariate analysis showed that the type of tumor pathology, FIGO stage, chemoresistance, HDL-C/LDL-C, HDL-C/TC, the presence of neoadjuvant chemotherapy and maintenance therapy were associated with progression free survival (PFS) and overall survival (OS) in patients with ovarian cancer. FIGO stage and chemoresistance were independent risk factors for FPS and OS. HDL-C/LDL-C ratio and post-operative maintenance therapy were independent protective factors for FPS. HDL-C/LDL-C ratio was also an independent protective factor for patients’ OS. Patients with higher HDL-C/LDL-C ratio have longer FPS and OS (p<0. 05). The mechanism may be that the conversion and metabolism of cholesterol within ovarian cancer cells affects cell proliferation, chemoresistance and metastasis, which in turn affects patient prognosis.

Our study shows that the HDL-C/TC and HDL-C/LDL-C ratios are closely related to the FIGO stage, chemoresistance and prognosis of ovarian cancer patients. Previous studies have suggested that statins such as lovastatin and Fluvastatin may play a potential role in cancer therapy strategy by modulating patients’ serum lipid levels. For example, statin can significantly reduce the risk of breast, colorectal, ovarian, and pancreatic cancer (32–35). In non-small cell lung cancer, lovastatin combined with gefitinib produces a synergistic anti-tumor effect, reducing cancer cell proliferation by inhibiting the epidermal growth factor receptor (36). Studies on ovarian cancer have found that fluvastatin and cisplatin can synergistically induce apoptosis of ovarian cancer cells by modulating Ras pathway (37). Based on our findings and relevant literature data, statins are expected to play an important role in the treatment of ovarian cancer in the future (38, 39).

This research is a single-center study with a relatively small sample size, so further multi-center, large-sample studies are needed to verify the relationship between serum lipids and clinical features and prognosis of ovarian cancer. We will conduct further molecular research on the relationship between lipid metabolism and biological behaviors of ovarian cancer cells and closely link them to patient’s serum lipid levels and prognosis, bringing new thinking to the treatment of ovarian cancer. Statins’ role in modulating lipid metabolism of tumor microenvironment therefore changing characteristics of cancer cells requires further investigation.

## Conclusion

In conclusion, serum lipid profiles are associated with the chemoresistance and prognosis of epithelial ovarian cancer. Higher HDL-C/LDL-C ratio and higher HDL-C/TC levels were protective factors for epithelial ovarian cancer, indicating novel index for screening and follow-up of ovarian cancer. At present, statins have been used in anti-tumor therapy. With the progress of research, lipid-lowering drugs are expected to play a more important role in improving the prognosis of patients.

## Data availability statement

The original contributions presented in the study are included in the article/supplementary material. Further inquiries can be directed to the corresponding author.

## Author contributions

Conceptualization: YL and HG; methodology: YL; software: YL; formal analysis: YL; writing - original draft: YL; all authors contributed to the article and approved the submitted version.
